# Relapsing Fever

**Published:** 1870-11

**Authors:** 


### Relapsing Fever.

*Philadelphia County Medical Society.*

Conversational Meeting, held September 28, 1870, at 8 p.m. Dr. Wm. H. Pancoast, President in the chair.

In reply to an inquiry as to the relapsing fever which has been prevalent in the lower section of the city, Dr. Welch (Attending physician of the Municipal Hospital) remarked, "Since I have had charge of the hospital, now abont one month, twenty-five cases of this fever have been admitted. During the summer some hundred of cases were treated by my predecessor. The vast majority have been from the southern section of the city, and from the squalid poor. As they present themselves, they are in all stages, from the inception of the fever to the relapse. We find, on inquiry, that they have been suddenly attacked with a chill, followed by fever. The first stage, or that of the first pyrexia, continues about one week, during which the pulse varies from 90 to 140. The tongue is heavily coated in its centre, with clean, red edges and tip. The coating is white and moist at first, but as the disease progresses it becomes yellowish, and sometimes brownish. I have rarely found well marked that clean triangular space at the end of the tongue, which has been described by some authors.

"A yellowish hue of the skin, and of the white of the eye, is sometimes observed in white patients; but in negroes I have found well-marked yellowness of the eyes uniformly present.

"Irritability of the stomach and epigastric tenderness are frequently seen. Tenderness over the region of the spleen is common. Muscular pain is a very frequent attendant, especially about the neck and shoulders.

"The skin is hot and dry at first, but for from twenty-four to forty-eight hours prior to the cessation of the pyrexia, a profuse perspiration generally occurs. The pulse now falls suddenly to its normal standard, or even below.

"This second stage, or intermission, continues about one week, the patient being free from fever; the tongue is clean, appetite returns, he walks about and believes himself convalescent. But on or about the fourteenth day from the onset of the disease, all the symptoms return, though in a milder form.

"The third stage, or relapse, does not last, on an average, more than three days. The intermission again occurs, and convalescence rapidly ensues. Another relapse may occur, but, in my experience, this is not frequent.

"The negro is much more profoundly impressed by the disease than the white, and the mortality is correspondingly greater. With my limited experience, I do not feel myself able as yet to diagnose the affection with certainty in its earliest stage. There are certain circumstances which may suggest the nature of disease, as the residence, the heavily coated tongue, with red tip and edges, and the muscular pain; but the relapse is, above all, the distinguishing symptom

"I have had two deaths, both negroes. In one an autopsy was held. He died under circumstances somewhat indicating yellow fever. A few hours before death, he vomited a thick grumous matter, and was without pulse at the wrist, though able to sit up. Soon this black vomit largely increased. Drs. E. Harris, of New York, and R. La Roche, of Philadelphia, were present at the examination. The liver was somewhat fatty, and was believed to be a "whiskey liver" The stomach was congested, and the spleen enlarged, weighing eleven ounces. This, with his history, and the absence of the lesions of the liver and stomach, found in yellow fever, sustained the diagnosis of relapsing fever.

"The treatment has been simple,—a febrifuge of solution of acetate of ammonia, with spirit of nitric ether and antimonial wine, turpentine, when the tongue was dry, and stimulants, if required. In the intermission, quinine only as a tonic. In a few cases, where there has been much jaundice, I have used calomel with benefit.

Dr. A. Douglass Hall inquired as to its resemblance to typhoid. He had seen one case in consultation, which at first sight reminded him of typhoid.

Dr, Welch had not been so impressed. The absence of diarhœa and nervous disorder, the typhoid tongue, iliac tenderness, and tympanites, and other symptoms of typhoid fever.

Dr. Atkin spoke of the differential diagnosis between relapsing fever and excluded the bilious remittent.

Dr. Welch.—"The most persistent bilious vomiting, and the remissions and exacerbations every day, or every other day, characterize bilious remittent, while relapsing fever pursues the course above detailed."

Dr. Buch has seen a number of cases in the southern part of the city. He had noticed violent headache, and great irritability of the stomach. The relapse occurred on the seventh and fourteenth days; in the latter case it was very severe. He paid great attention, employing injections of beef tea, with pepsin and dilute muriatic acid, when the stomach was very irritable. He relied greatly on quinia. He had lost no cases. In conversation with Drs. W. Pepper and Shapleigh, he had been informed that they found small yellowish deposits in the parenchyma of different organs.

In this connection he would mention a singular case, which proved not to be relapsing fever, where at twelve o'clock, nearly every night, the patient, a boy seven years old, became insane; the insanity would pass off in the morning. At the end of two weeks death ensued. The bowels were regular, appetite good, no cough, to tympanites. He thought it a case of malarial fever.

Dr. Atkinson, though constantly attending cases throughout the infected district, had not seen a case of true relapsing fever. He had encountered several cases of fever which presented symptoms of such an attack, but were of an ephemeral nature, none lasting more than two or three days, and none suffering a relapse.

He mentioned a case which had just occurred in close proximity to the recent cases of yellow fever. It was marked by profuse epistaxis, frequently renewed, intense cephalalgia, great muscular pain and prostration, but with complete intermissions in the febrile movement. The patient rapidly convalesced under the exhibition of antiperiodic doses of quinine.

Dr. Fish narrated two cases, which tended to establish the contagious character of relapsing fever. He would ask Dr. Welch if his experience and observation at the Municipal Hospital had confirmed the contagiousness of the disease.

Dr. Welch had seen no case directly traceable to contagion.

Dr. Wittig made some remarks in regard to the fact that in many morbid conditions there is a tendency to occurrence of a relapse, soon after the primary attack, on re-exposure to the exciting cause.—*Philadelphia Medical Times*.

